# Dysregulation of lncRNA‐CCRR contributes to brain metastasis of breast cancer by intercellular coupling via regulating connexin 43 expression

**DOI:** 10.1111/jcmm.16455

**Published:** 2021-04-01

**Authors:** Deheng Li, Liangdong Li, Xin Chen, Changshuai Zhou, Bin Hao, Yiqun Cao

**Affiliations:** ^1^ Department of Neurosurgery Fudan University Shanghai Cancer Center Shanghai China; ^2^ Department of Oncology Shanghai Medical College Fudan University Shanghai China

**Keywords:** astrocyte, breast cancer metastasis to brain, connexin 43, gap junction, lncRNA‐CCRR

## Abstract

Cardiac conduction regulatory RNA (CCRR) is down‐regulated in the pathogenesis of heart failure (HF), which accordingly suppresses cardiac conduction while promoting arrhythmogenicity. Meanwhile, CX43 was reported to play a role in the pathogenesis of metastatic breast cancer and melanoma brain colonization. In this study, we studied the role of long non‐coding RNA CCRR and its interaction with CX43 in brain metastasis of breast cancer. Breast cancer patients were grouped according to the metastasis status. Real‐time PCR and IHC assay were used to measure the expression of lncRNA‐CCRR and CX43 in patients. Western blot was conducted to observe the effect of lncRNA‐CCRR on the expression of CX43 in MDA‐MB‐231BR and BT‐474BR cells. Compared with the non‐metastasis group, the mRNA expression of tissue lncRNA‐CCRR, cerebrospinal fluid (CSF) lncRNA‐CCRR, tissue CX43 and tissue protein expression of CX43 were both evidently up‐regulated in metastasis patients, especially in patients with brain metastasis. The expression of lncRNA‐CCRR was positively correlated with the up‐regulated expression of CX43. Moreover, CX43 expression was significantly lower in MDA‐MB‐231WT cells compared with that in MDA‐MB‐231BR cells. Also, the overexpression of lncRNA‐CCRR evidently increased dye transfer rate from astrocytes to MDA‐MB‐231BR/BT‐474BR cells but reduced lncRNA‐CCRR expression and suppressed the transmigration of MDA‐MB‐231BR/BT‐474BR cells in a blood‐brain barrier (BBB) model. In this study, we demonstrated that the presence of lncRNA‐CCRR could up‐regulate the expression of CX43, which promoted gap junction formation in brain metastasis of breast cancer. Accordingly, the communication between breast cancer cells and astrocytes was also promoted.

## 1 INTRODUCTION

About 10% of patients with cancer tend to develop brain metastasis.^1^, ^2^, ^3^ Also, small lesions of brain metastasis still can trigger nerve impairment, so that the average survival of brain metastasis patients is short,^1^ and the two primary reasons of brain metastasis are lung adenocarcinomas and breast adenocarcinomas.^2^ Lung adenocarcinoma metastasis tends to develop within months of medical diagnosis to exert an effect on a number of organs outside the brain, suggesting that aggressive pre‐metastatic functions will foster cancer cell colonization at different organs at the same time.^4^ In breast cancer, distant relapse often occurs after an extended period of remission, indicating that breast cancer cells do not have the total capability for metastasis initially in distant organs.^5^, ^6^ Instead, breast cancer cells obtain this capability for metastasis under the selective stress pressure of microenvironments in various organs.^1^

Lately, long non‐coding RNA (lncRNA) has actually emerged as a key player in the regulation of gene expression.^7^, ^8^ LncRNAs are also recognized as regulatory molecules featured by lack of protein‐coding capability. However, lncRNAs may take part in numerous vital biological processes as well as pathophysiological activities.^9^, ^10^ As the first lncRNA determined to show the ability to manage cardiac conduction, lncRNA AK045950 is also called cardiac conduction regulatory RNA (CCRR), which is actually down‐regulated in a heart failure (HF) mouse model as well as people with HF by slowing down cardiac conduction as well as enhancing arrhythmogenicity.^11^

However, CCRR was needed for keeping appropriate connexin 43 (CX43) distribution in intercalated discs and prevent the backward trafficking as well as subsequent degradation of CX43, a mechanism likely acted to disrupt gap junctions.^11^

CX43, as a gap junction channel protein, was additionally discovered to become dysregulated in various forms of cancers such as stomach, cervical, rectal and prostate cancers. In breast cancer cells, the down‐regulation of CX43 substantially enhanced cancer cell growth, while CX43 overexpression possibly suppressed cancer cell growth and restored their differentiation potential to suppress tumour.^12^ In addition, the ubiquitylation of CX43 resulted in gap junction accumulation at cell membrane to trigger a concomitant boost of intercellular interaction.^13^, ^14^ The brain is a big target of metastasis, with astrocytes acting as the predominant mediator.

The function of astrocytes, most abundant type of cells in the brain, has been studied in brain metastasis. Breast as well as lung cancer cells can express PCDH7 to promote the assembly of astrocytes‐cancer cell gap junctions comprised of CX43. After engaging the gap junction network in astrocytes, cancer cells metastasizing to brain utilize these gap junctions to transfer cGAMP into astrocytes, activating the STING signalling as well as the synthesis of proinflammatory cytokines IFNα and TNFα.^15^ It has been reported that CCRR is down‐regulated in a mouse model of HF and in patients with HF, and this down‐regulation slows cardiac conduction and enhances arrhythmogenicity.^11^ Meanwhile, CX43 was reported to play a role in the pathogenesis of metastatic breast cancer and melanoma brain colonization.^16^ In this study, we hypothesized that lncRNA‐CCRR could interact with CX43 and regulate its expression, and the dysregulated CX43 influenced gap junction formation in brain metastasis of breast cancer, which accordingly influenced the communication between breast cancer cells and astrocytes.

## 2 MATERIALS AND METHODS

### 2.1 Patient recruitment

In this study, 64 breast cancer patients were grouped to the following three groups according to their metastasis status: (1) brain metastasis (‐)/other metastasis (‐) (N = 24) [breast cancer patients with neither brain metastasis nor other metastasis]; (2) brain metastasis (‐)/other metastasis (+) (N = 22) [breast cancer patients with no brain metastasis but with other metastasis]; (3) brain metastasis (+)/other metastasis (+) (N = 18) [breast cancer patients with both brain metastasis and other metastasis]. These patients were recruited from January 2017 to December 2018 at Fudan University Shanghai Cancer Center. The mean age of these patients were 53.73 and all patients were female. Institutional ethical committee has approved the protocol of this study. Written informed consent was obtained from each patient before the study.

### 2.2 Cell culture and transfection

MDA‐MB‐231BR and BT‐474BR were both brain metastatic variant of MDA‐MB‐231 cell line and BT‐474 cell line, respectively. In this study, the MDA‐MB‐231BR and BT‐474BR cells were obtained from Lonza and affirmed to be free of mycoplasma. The culture of MDA‐MB‐231BR and BT‐474BR cells was done under standard cell culture environment of 37°C, 5% CO_2_ and 95% air. The cell culture medium was DMEM (Lonza) added with 10% heat inactivated FBS and penstrep.

In this study, different cell models were created.

In cell model I, MDA‐MB‐231BR and BT‐474BR cells were divided into two groups: (1) NC group [MDA‐MB‐231BR and BT‐474BR cells transfected with a negative control] and (2) lncRNA‐CCRR group [MDA‐MB‐231BR and BT‐474BR cells transfected with lncRNA‐CCRR].

In cell model II, MDA‐MB‐231BR and BT‐474BR cells were also divided into two groups: (1) NC siRNA group [MDA‐MB‐231BR and BT‐474BR cells transfected with a NC siRNA] and (2) lncRNA‐CCRR siRNA group [MDA‐MB‐231BR and BT‐474BR cells transfected with lncRNA‐CCRR siRNA].

In cell model III, MDA‐MB‐231BR cells were divided into four groups: (1) MDA‐MB‐231WT group [wild‐type MDA‐MB‐231 cells]; (2) MDA‐MB‐231BR group [untreated MDA‐MB‐231BR cells]; (3) MDA‐MB‐231BR + NC siRNA group [MDA‐MB‐231BR cells transfected with a NC siRNA]; and (4) MDA‐MB‐231BR + lncRNA‐CCRR siRNA group [MDA‐MB‐231BR cells transfected with lncRNA‐CCRR siRNA].

In cell model IV, BT‐474BR cells were divided into four groups: (1) BT‐474WT group [wild‐type BT‐474BR cells]; (2) BT‐474BR group [untreated BT‐474BR cells]; (3) BT‐474BR + NC siRNA group [BT‐474BR cells transfected with a NC siRNA]; and (4) BT‐474BR + lncRNA‐CCRR siRNA group [BT‐474BR cells transfected with lncRNA‐CCRR siRNA].

In cell model V, MDA‐MB‐231BR and BT‐474BR cells were divided into three groups: (1) NC group [MDA‐MB‐231BR and BT‐474BR cells transfected with a negative control]; (2) co‐astrocyte + NC siRNA group [MDA‐MB‐231BR and BT‐474BR cells co‐cultivated with astrocytes and transfected with a NC siRNA]; (3) co‐astrocyte + lncRNA‐CCRR siRNA group [MDA‐MB‐231BR and BT‐474BR cells co‐cultivated with astrocytes and transfected with lncRNA‐CCRR siRNA].

All cell transfection was carried out using Lipofectamine 2000 (Invitrogen) following the standard transfection protocol provided by the transfection reagent manufacturer. The transfected cells were harvested 24 hours after transfection to analyse target gene expression.

### 2.3 RNA isolation and real‐time PCR

RNA isolation, reverse transcription of isolated RNA and real‐time polymerase chain reaction (RT‐PCR) were carried out conventionally to measure the expression of lncRNA‐CCRR and CX43 mRNA in each sample. In brief, total RNA was isolated by making use of a TRIzol reagent (Invitrogen) following the standard experimental protocol provided on the operation manual by the assay kit manufacturer. For mRNA quantification, the total RNA was reverse transcribed by utilizing a PrimeScript RT Reagent assay kit (TaKaRa) following the standard experimental protocol provided on the operation manual by the assay kit manufacturer. In the next step, the RT‐PCR was conducted by making use of SYBR Green Master Mix (Applied Biosystems) on an ABI 7500 RT‐PCR system (Applied Biosystems) following the standard experimental protocol provided on the operation manual by the assay kit manufacturer. The relative gene expression of lncRNA‐CCRR (forward: 5′‐GACTGAGCTTTGAAAATATG‐3′; reverse: 5′‐GTCCCATCCCCAAGCTGCTTGATC‐3′) and CX43 mRNA (forward: 5′‐TGTAAAACGACGGCCAGT‐3′; reverse: 5′‐CAGGAAACAGCTATGACC‐3′) in each sample was determined based on the 2^−ΔΔCT^ strategy, while GAPDH (forward: 5′‐ GTCTCCTCTGACTTCAACAGCG‐3′; reverse: 5′‐ACCACCCTGTTGCTGTAGCCAA‐3′) was used as the calibrator.

### 2.4 Western blot analysis

Western blot analysis was carried out to measure the protein expression of CX43 in each sample. In brief, total protein was isolated from cell and tissue samples by making use of a radioimmunoprecipitation (RIPA) buffer (Thermo Fisher Scientific) following the standard experimental protocol provided on the operation manual by the buffer manufacturer. Then, the concentration of protein in each sample was determined by utilizing a bicinchoninic acid (BCA) assay kit (Thermo Fisher Scientific) following the standard experimental protocol provided on the operation manual by the assay kit manufacturer. In the next step, the proteins were resolved by using 10% sodium dodecyl sulphate‐PAGE and then transferred onto a polyvinylidene difluoride membrane via electroblotting. In the next step, non‐specific binding was blocked with Tris‐buffered saline with Tween‐20 + 5% non‐fat milk before the membrane was probed overnight at 4°C with primary anti‐CX43 antibody (dilution 1:1000, OriGene Technologies) following the standard incubation protocol provided on the operation manual by the antibody manufacturer, followed by subsequent incubation with HRP‐conjugated secondary antibodies (dilution 1:5000, Thermo Fisher Scientific) for 1 hour at ambient temperature. After image development by making use of an enriched chemiluminescence assay kit (Thermo Fisher Scientific) following the standard experimental protocol provided on the operation manual by the assay kit manufacturer, the protein expression of CX43 in each sample was calculated.

### 2.5 IHC

Immunohistochemical staining was done to visualize the protein expression of CX43 in each sample. In brief, tissue samples were fixed in formalin, embedded in paraffin, deparaffinized and then hydrated by utilizing gradient alcohol. The samples were then blocked in PBS + 0.2% Triton X‐100 + 10% horse serum for 1 hour at room temperature. After that, the samples were incubated against primary anti‐CX43 antibodies and biotin conjugated secondary antibodies following the standard incubation protocol provided on the operation manual by the antibody manufacturer (Dako). Dab was used as the dye for counter‐staining.

### 2.6 Dye transfer assay

Due to the fact that the communication between non‐tumour astrocytes and breast carcinoma cells influences brain metastasis, the effect of lncRNA‐CCRR on dye transfer rate was investigated via the dye transfer analysis. The dye transfer assay was done using a calcein‐AM/DiD assay kit (Takara) following the standard experimental protocol provided on the operation manual by the assay kit manufacturer. The results were evaluated on a FACS Canto II Flow Cytometer (BD Biosciences) and analysed using the FlowJo software (Treestar).

### 2.7 Transwell assay

To observe the effect of lncRNA‐CCRR expression on the transmigration in an organotypic BBB model established based on co‐cultivation of PBECs with astrocytes, a transwell assay was performed on MDA‐MB‐231BR cells, respectively, grouped as: (1) negative control group; (2) co‐astrocyte + NC siRNA group; and (3) co‐astrocyte + lncRNA‐CCRR siRNA group. The transwell assay was performed using the BBB model co‐culture Transwell system (Thermo Fisher Scientific) following the standard experimental protocol provided on the operation manual by the transwell manufacturer. The TEER value was gauged by using an EVOM epithelial Volt/Ohm (TEER) Meter (World Precision Instruments) following the standard experimental protocol provided on the operation manual by the instrument manufacturer. The established model had a TEER value of >200 × cm^2^.

### 2.8 Statistical analysis

The statistical analysis was carried out using the SPSS software package (version 19.0, SPSS). The data was shown as mean ± SD. One‐way analysis of difference (ANOVA) was done to compare the difference among different groups. A Student's *t* test was done to compare the difference between two groups. A value of *P* < .05 suggested statistical significance.

## 3 RESULTS

### 3.1 Expression of lncRNA‐CCRR and CX43 was higher in patients with metastasis

**FIGURE 1:**
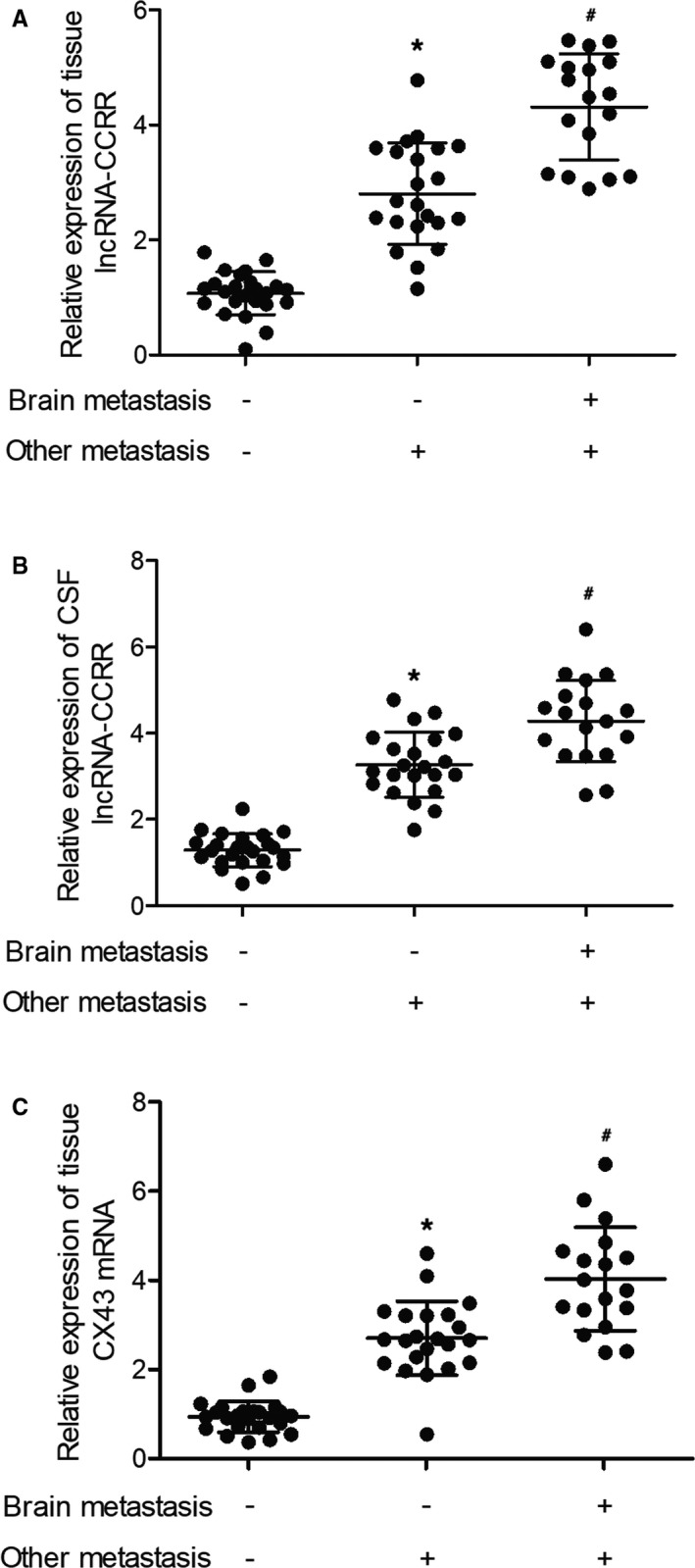
Expression of lncRNA‐CCRR and CX43 was higher in patients with metastasis. A, Expression of tissue lncRNA‐CCRR in different patient groups (* *P* < .05 compared with brain metastasis (‐)/other metastasis (‐) group; # *P* < .05 compared with brain metastasis (‐)/other metastasis (+) group). B, Expression of CSF lncRNA‐CCRR in different patient groups (* *P* < .05 compared with brain metastasis (‐)/other metastasis (‐) group; # *P* < .05 compared with brain metastasis (‐)/other metastasis (+) group); C: Expression of tissue CX43 mRNA in different patient groups (* *P* < .05 compared with brain metastasis (‐)/other metastasis (‐) group; # *P* < .05 compared with brain metastasis (‐)/other metastasis (+) group)

**FIGURE 2:**
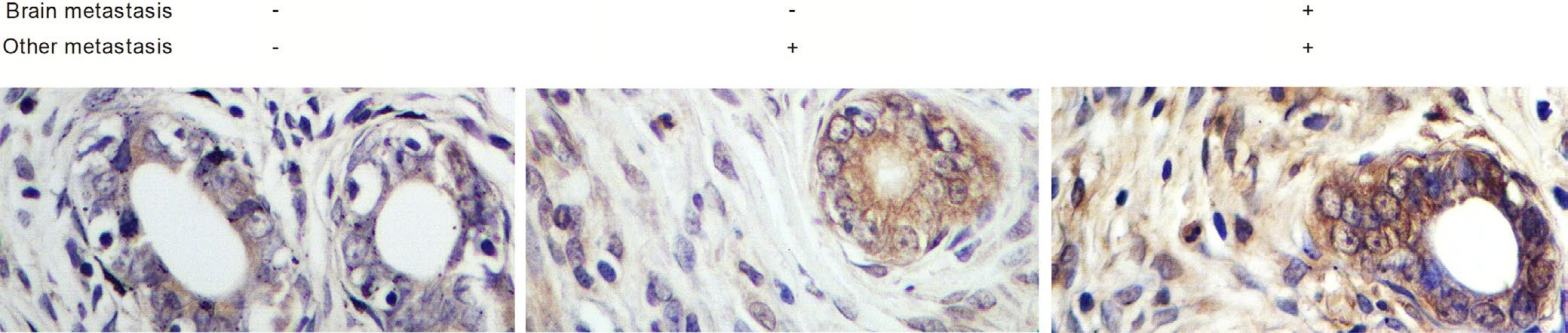
IHC assay indicated that the expression of tissue CX43 protein was evidently up‐regulated in metastasis patients, especially in patients with brain metastasis

The breast cancer patients were grouped according to their metastasis status as: (1) brain metastasis (‐)/other metastasis (‐) (N = 24); (2) brain metastasis (‐)/other metastasis (+) (N = 22); (3) brain metastasis (+)/other metastasis (+) (N = 18). The real‐time PCR was performed to measure the expression of lncRNA‐CCRR in tissue and CSF samples collected from different patient groups. Accordingly, compared with those in the non‐metastasis group, tissue expression of lncRNA‐CCRR (Figure 1A) and CSF expression of lncRNA‐CCRR (Figure 1B) were both evidently up‐regulated in metastatic patients, while the patients with brain metastasis showed even higher lncRNA‐CCRR expression compared with patients with no brain metastasis. Also, the relative expression of CX43 mRNA in tissues (Figure 1C) and CX43 protein in tissues (Figure 2) also exhibited the same trend.

### 3.2 Overexpression of lncRNA‐CCRR promoted the expression of CX43

**FIGURE 3:**
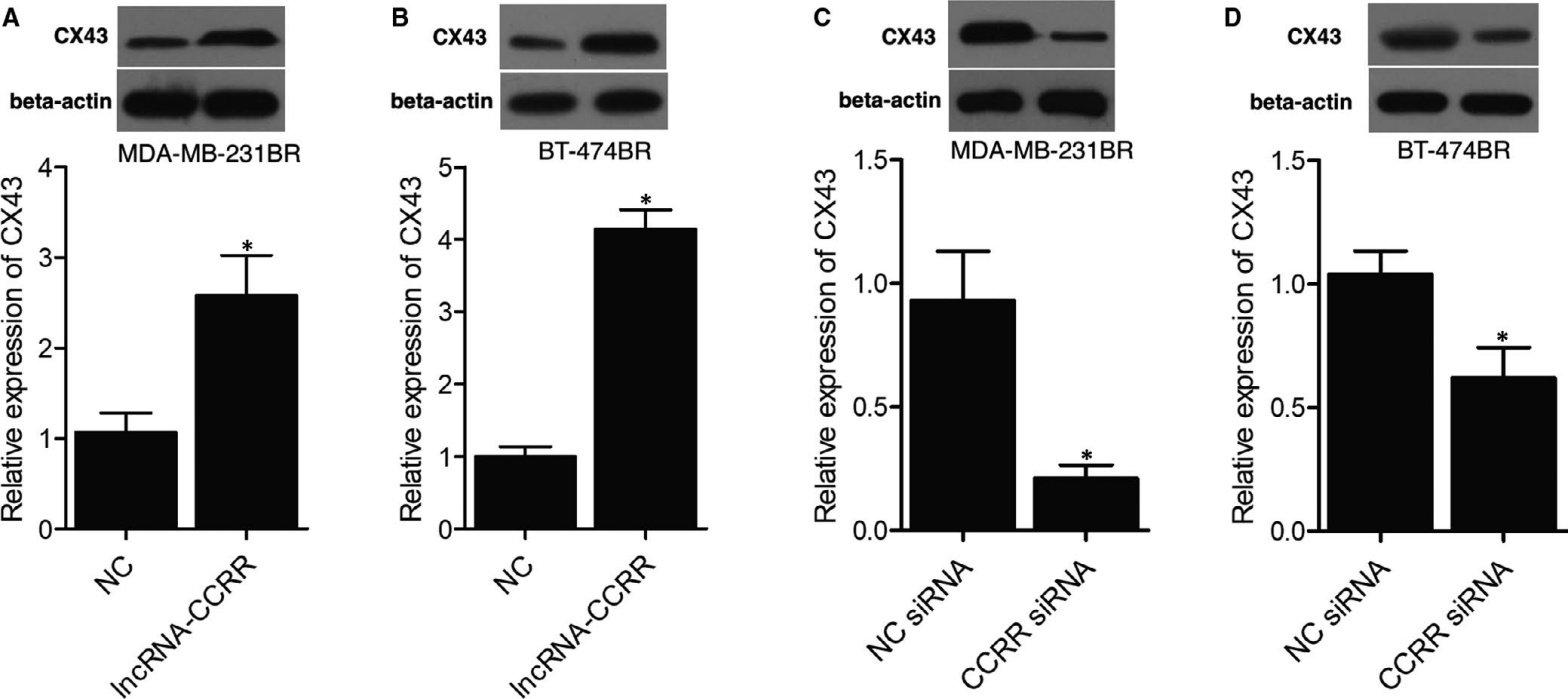
Overexpression of lncRNA‐CCRR promoted the expression of CX43. A, Expression of CX43 in MDA‐MB‐231BR cells transfected with lncRNA‐CCRR compared with that in MDA‐MB‐231BR cells transfected with a negative control (* *P* < .05 compared with NC group). B, Expression of CX43 in BT‐474BR cells transfected with lncRNA‐CCRR compared with that in BT‐474BR cells transfected with a negative control (* *P* < .05 compared with NC group). C, Expression of CX43 in MDA‐MB‐231BR cells transfected with lncRNA‐CCRR siRNA compared with that in MDA‐MB‐231BR cells transfected with NC siRNA (* *P* < .05 compared with NC siRNA group). D, Expression of CX43 in BT‐474BR cells transfected with lncRNA‐CCRR siRNA compared with that in BT‐474BR cells transfected with NC siRNA (* *P* < .05 compared with NC siRNA group)

To study the effect of lncRNA‐CCRR on the expression of CX43, MDA‐MB‐231BR cells were transfected with lncRNA‐CCRR or lncRNA‐CCRR siRNA. Accordingly, compared with MDA‐MB‐231BR cells in the NC group, the relative expression of CX43 was up‐regulated in lncRNA‐CCRR‐transfected MDA‐MB‐231BR cells (Figure 3A). Moreover, compared with MDA‐MB‐231BR cells transfected with NC siRNA, the transfection of lncRNA‐CCRR siRNA significantly inhibited the expression of CX43 (Figure 3C). When the above observation was repeated in BT‐474BR cells, similar results were also obtained (Figure 3B,D).

### 3.3 Expression of CX43 was increased in cells with brain metastasis

**FIGURE 4:**
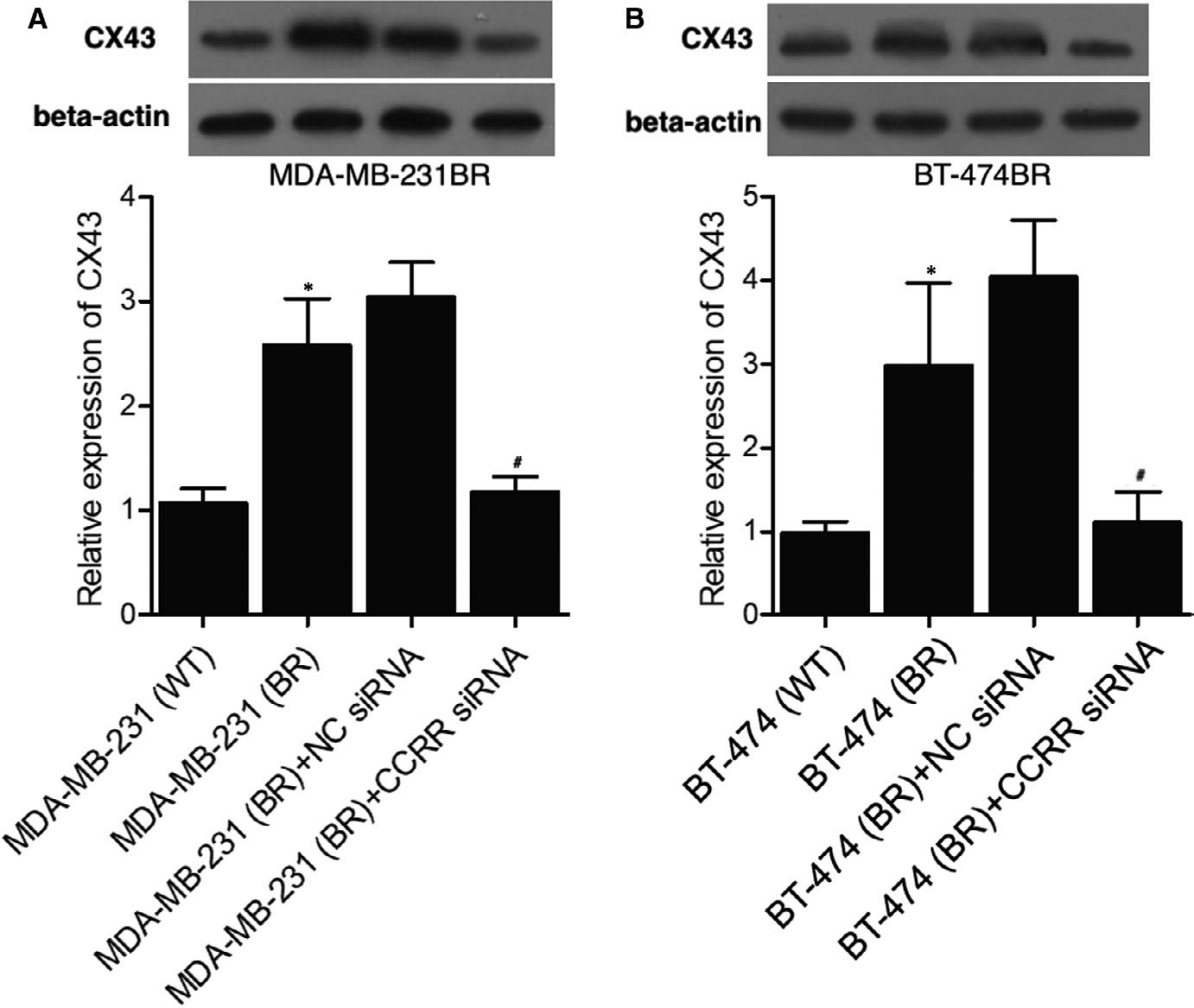
Expression of CX43 was increased in cells with brain metastasis. A, Expression of CX43 in the MDA‐MB‐231WT group, MDA‐MB‐231BR group, MDA‐MB‐231BR + NC siRNA group and MDA‐MB‐231BR + lncRNA‐CCRR siRNA group (* *P* < .05 compared with MDA‐MB‐231WT group; # *P* < .05 compared with MDA‐MB‐231BR + NC siRNA group). B, Expression of CX43 in the BT‐474WT group, BT‐474BR group, BT‐474BR + NC siRNA group and BT‐474BR + lncRNA‐CCRR siRNA group (* *P* < .05 compared with BT‐474WT group; # *P* < .05 compared with BT‐474BR + NC siRNA group)

To further explore the influence of brain metastasis on the effect of lncRNA‐CCRR, MDA‐MB‐231WT cells were utilized as the control group to compare with MDA‐MB‐231BR cells. As indicated by the Western blot results (Figure 4A), the relative expression of CX43 was significantly lower in MDA‐MB‐231WT cells compared with that in MDA‐MB‐231BR cells, and the CX43 expression was comparable between MDA‐MB‐231 BR and MDA‐MB‐231BR + NC siRNA groups, but the transfection of lncRNA‐CCRR siRNA down‐regulated lncRNA‐CCRR and CX43 expression. Moreover, when the above observation was repeated BT‐474WT and BT‐474BR cells, similar results were obtained (Figure 4B).

### 3.4 Overexpression of lncRNA‐CCRR inhibited cell communication between non‐tumour astrocytes and breast carcinoma cells

**FIGURE 5:**
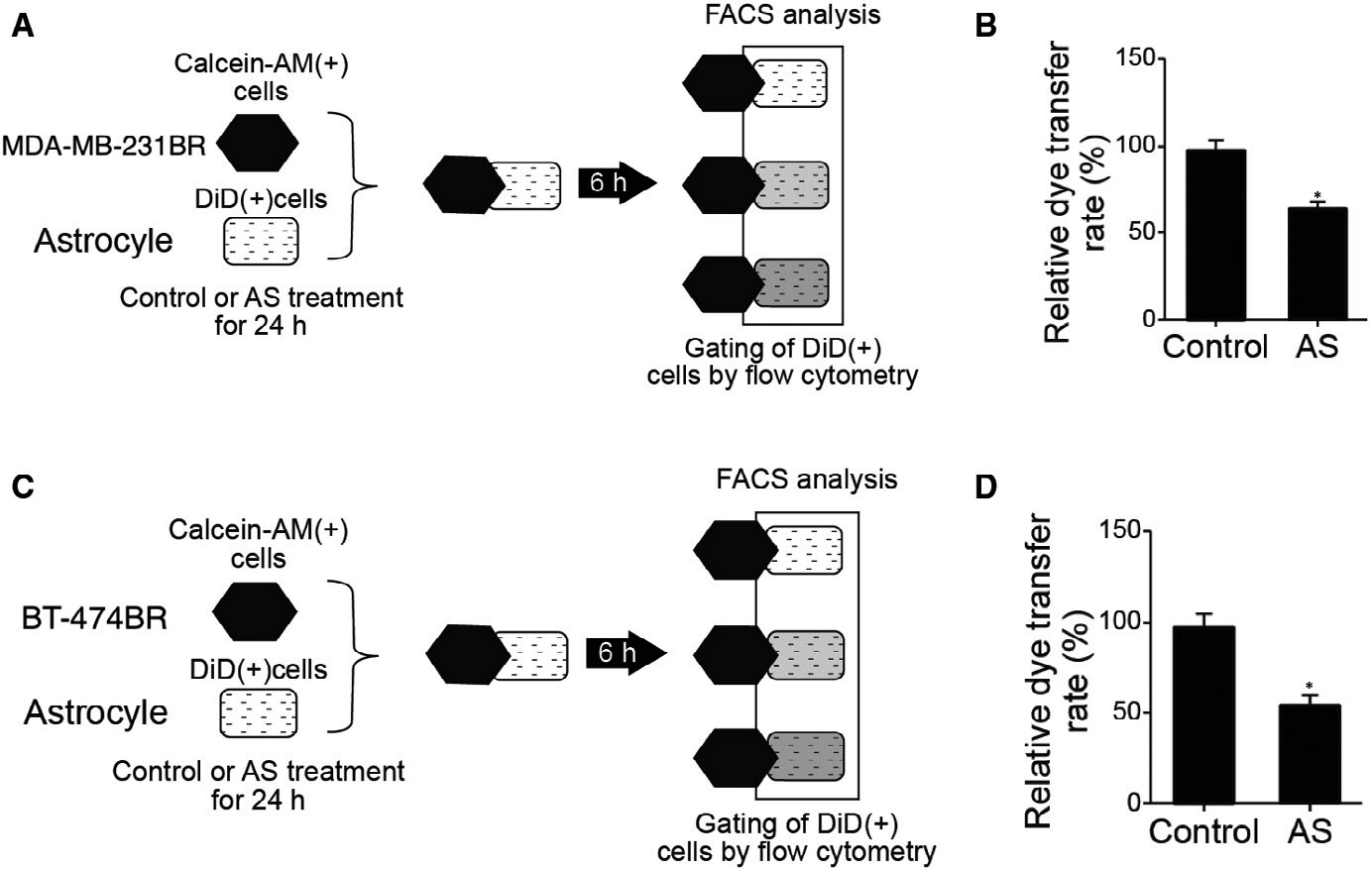
Overexpression of lncRNA‐CCRR inhibited the communication between non‐tumour astrocytes and breast carcinoma cells. A, Dye transfer analysis of astrocyte‐MDA‐MB‐231BR communication. B, Histograms and quantification of dye transfer between astrocytes and MDA‐MB‐231BR cells (* *P* < .05 compared with control group). C, Dye transfer analysis of astrocyte‐BT‐474BR communication; D, Histograms and quantification of dye transfer between astrocytes and BT‐474BR cells (* *P* < .05 compared with control group)

Due to the fact that the communication between non‐tumour astrocytes and breast carcinoma cells influences brain metastasis, the effect of lncRNA‐CCRR on dye transfer rate was investigated via the dye transfer analysis. As shown in Figure 5A,B, the overexpression of lncRNA‐CCRR evidently increased the dye transfer rate from astrocytes to MDA‐MB‐231BR cells, and the overexpression of lncRNA‐CCRR also increased the dye transfer rate from astrocytes to BT‐474BR cells (Figure 5C,D), indicating that lncRNA‐CCRR could promote cell communication between breast carcinoma cells and non‐tumour astrocytes.

### 3.5 Reduced lncRNA‐CCRR expression suppressed transmigration in a BBB model

**FIGURE 6:**
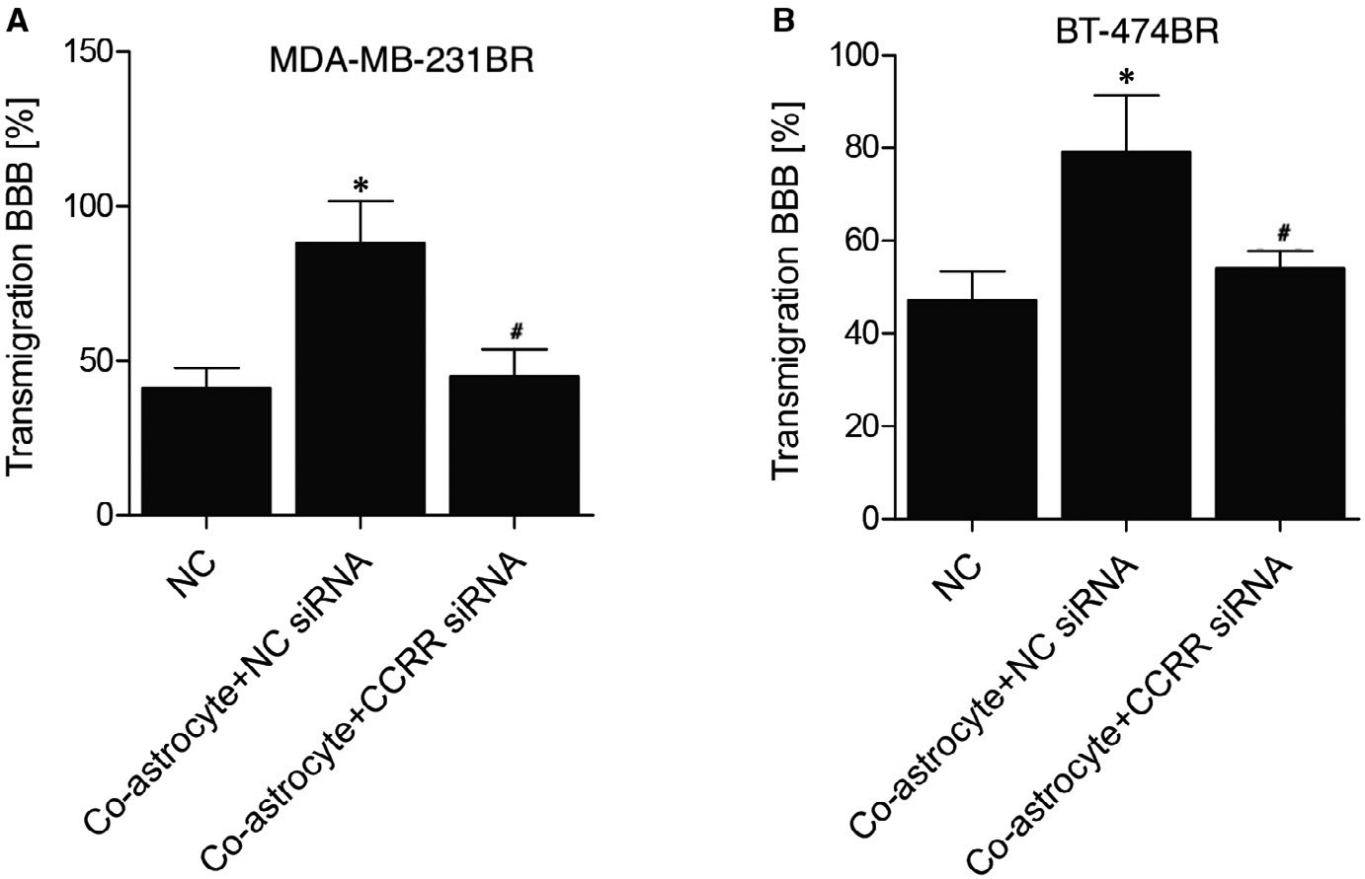
Reduced lncRNA‐CCRR expression suppressed the transmigration in a BBB model. A, Quantification of the transmigration BBB (%) of MDA‐MB‐231BR cells (* *P* < .05 compared with NC group; # *P* < .05 compared with co‐astrocyte + NC siRNA group). B, Quantification of the transmigration BBB (%) of BT‐474BR cells (* *P* < .05 compared with NC group; # *P* < .05 compared with co‐astrocyte + NC siRNA group)

To observe the effect of lncRNA‐CCRR expression on the transmigration in an organotypic BBB model established based on co‐cultivation of PBECs with astrocytes, as shown in Figure 6A, A transwell assay was performed on the MDA‐MB‐231BR cell groups, and the according quantification of the transmigration BBB (%) indicated that the reduced expression of lncRNA‐CCRR suppressed the elevated transmigration of MDA‐MB‐231BR cells in the BBB model. Also, similar results were demonstrated in BT‐474BR cells (Figure 6B).

### 3.6 Reduced lncRNA‐CCRR expression suppressed the expression of cellular CX43

**FIGURE 7:**
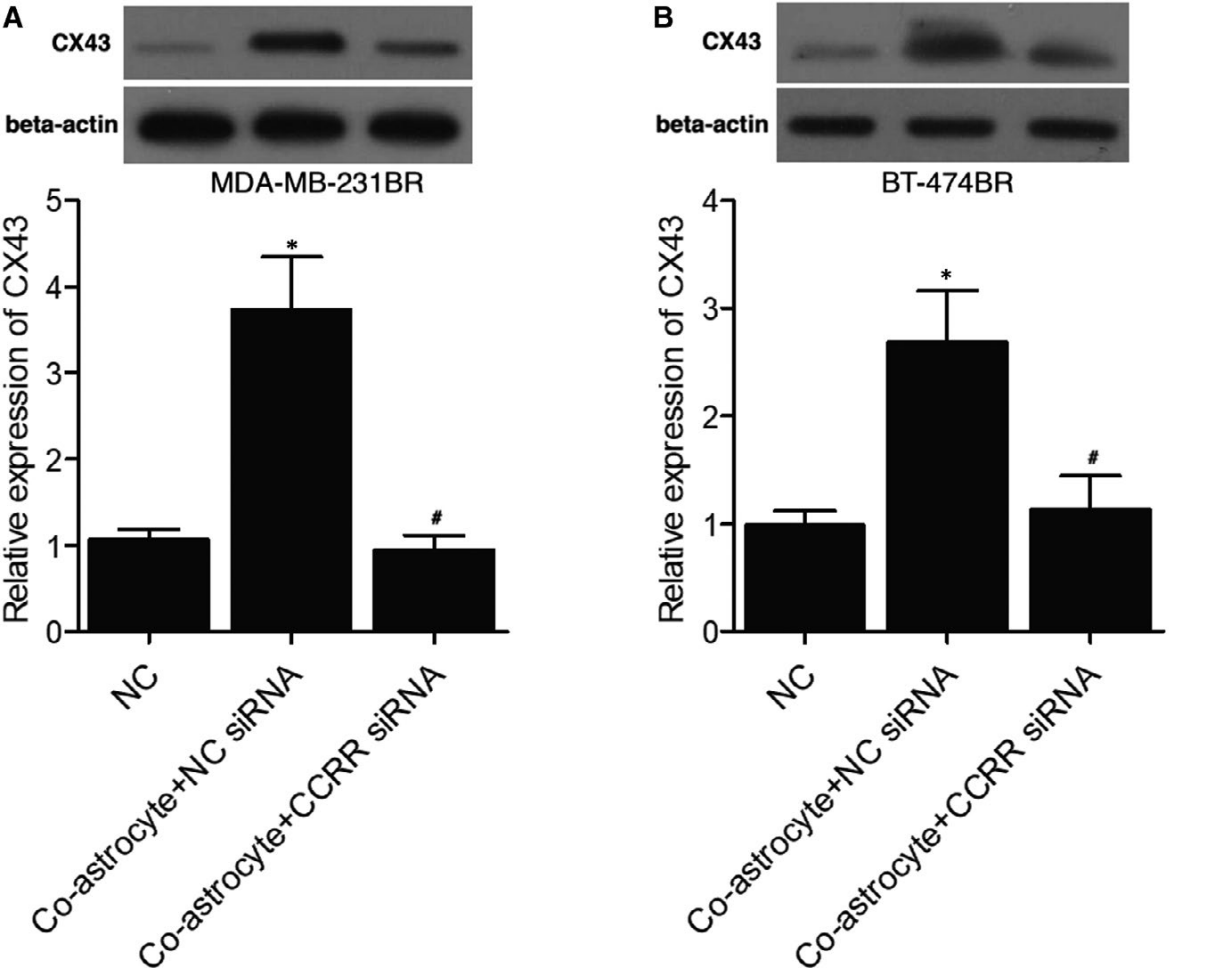
Reduced lncRNA‐CCRR expression suppressed the expression of cellular CX43. A, Expression of CX43 among different MDA‐MB‐231BR cell groups (* *P* <.05 compared with NC group; # *P* < .05 compared with co‐astrocyte + NC siRNA group). B, Expression of CX43 among different BT‐474BR cell groups (* *P* < .05 compared with NC group; # *P* < .05 compared with co‐astrocyte + NC siRNA group)

The levels of CX43 among different MDA‐MB‐231BR (Figure 7A) and BT‐474BR cell groups (Figure 7B) were also compared. The expression of CX43 was significantly elevated in the co‐astrocyte + NC siRNA group compared with that in the negative control group, while the down‐regulation of lncRNA‐CCRR by the transfection of lncRNA‐CCRR siRNA reversed the deregulation of CX43 in MDA‐MB‐231BR cells or BT‐474BR cells.

## 4 DISCUSSION

In this study, we collected tissue samples from breast cancer patients with or without brain metastasis and found that, compared with that in the non‐metastasis group, the expression of tissue lncRNA‐CCRR, CSF lncRNA‐CCRR, tissue CX43 mRNA and tissue CX43 protein was all evidently up‐regulated in metastasis patients, while patients with brain metastasis showed even higher lncRNA‐CCRR expression compared with patients with no brain metastasis. A previous report showed that a CCRR sequence is conserved in many species and is responsible for the CCRR‐CX43 interacting protein of 85 kDa (CIP85) communication as well as the subsequent disruption in the CIP85‐CX43 interaction, which is enough to generate the beneficial action of full‐length CCRR. It was also concluded that CCRR acts as an anti‐arrhythmic lncRNA via maintaining the regular myocardial syncytium.^11^ Thereby, CCRR may bind to CIP85 to interfere with the CIP85‐CX43 interaction. The theoretical analyses of RNA‐protein binding with the RPISeq database also uncovered a higher probability of CCRR‐CIP85 interaction, showing evidence that CCRR can bind to CIP85 to ultimately reduce the CX43‐CIP85 interaction.^11^ In this study, we found that, compared with the untreated breast cancer cells, the expression of CX43 was up‐regulated in cells transfected with lncRNA‐CCRR. Moreover, compared with the breast cancer cells transfected with NC siRNA, the transfection of lncRNA‐CCRR siRNA significantly inhibited the expression of CX43.

One way by which the cells coordinate their functions is through cell to cell communication carried out via the gap junction protein.^17^, ^18^ As the most abundant gap junction protein present in the skeletal system, CX43 deletion significantly reduced bone mass and induced osteoblast dysfunction.^19^, ^20^, ^21^ In this study we found that the relative expression of CX43 was significantly lower in MDA‐MB‐231WT cells compared with that in MDA‐MB‐231BR cells, and the CX43 expression was comparable between MDA‐MB‐231 BR and MDA‐MB‐231BR + NC siRNA groups, but the transfection of lncRNA‐CCRR siRNA down‐regulated the expression of lncRNA‐CCRR and CX43. Similar results were observed in BT‐474BR cells.

Connexins were shown to display tumour suppressor activities to induce the dedifferentiation of cancer cells, eventually preventing metastasis.^22^, ^23^, ^24^ Furthermore, mice with inactivated CX43 showed delayed occurrence of palpable mammary tumours by showed increased metastases in the lung.^25^ Also, metastatic breast cancer cells showed an enhanced level of CX43 expression, and only the breast cancer cells that were positive for CX43 could colonize in adjacent proximity to the vascular tissues in human brain.^16^, ^26^

CX43 is found in metastatic tumour cells at the communication interface with astrocytes.^20^ The past results showed that the capacity of cancer stem cells (CSCs) to metastasis to the brain may be triggered by the higher CX43 expression in CSCs. Furthermore, it was recently shown that the transfer of cGAMP to astrocytes from cancer cells through CX43‐containing enhances the brain metastasis of cancer, suggesting that the gap junctions between astrocytes and tumour cells can be used as a good target in the treatment of metastatic cancer.^15^, ^27^ In this study, we found that the overexpression of lncRNA‐CCRR evidently increased the dye transfer rate from astrocytes to MDA‐MB‐231BR/ BT‐474BR cells. Furthermore, the transmigration BBB (%) indicated that the reduced expression of lncRNA‐CCRR suppressed the elevated transmigration of MDA‐MB‐231BR/BT‐474BR cells in the BBB model. Moreover, the expression of CX43 was significantly elevated in the co‐astrocyte + NC siRNA group, while the down‐regulation of lncRNA‐CCRR by the transfection of lncRNA‐CCRR siRNA reversed the deregulation of CX43 in MDA‐MB‐231BR cells or BT‐474BR cells. Recently, it was reported that the CX43‐containing gap junctions are the key constituent in astrocytes as well as epithelial lung cancer cells to enhance the cancer metastasis to brain via transferring cGAMP to astrocytes.^15^, ^28^, ^29^, ^30^

## 5 CONCLUSION

In this study, we demonstrated that the presence of lncRNA‐CCRR could up‐regulate the expression of CX43, which promoted gap junction formation in brain metastasis of breast cancer. Accordingly, the communication between breast cancer cells and astrocytes was also promoted.

## CONFLICT OF INTEREST

None.

## AUTHOR CONTRIBUTION

**Deheng Li :** Conceptualization (lead); Methodology (equal); Resources (equal); Validation (equal); Writing‐original draft (lead). **Liangdong Li:** Conceptualization (equal); Investigation (equal); Software (equal); Visualization (equal). **Xin Chen:** Data curation (equal); Formal analysis (equal); Investigation (equal); Software (equal). **Changshuai Zhou:** Conceptualization (equal); Data curation (equal); Software (equal); Validation (equal). **Bin Hao:** Data curation (equal); Investigation (equal); Software (equal); Visualization (equal). **Yiqun Cao:** Investigation (equal); Methodology (equal); Project administration (lead); Resources (equal); Supervision (equal); Writing‐review & editing (lead).

## Data Availability

The data that support the findings of this study are available from the corresponding author upon reasonable request.
